# Improving Education and Training to Reduce the Burden of Occupational Cancer. The Riga-European Association of Schools of Occupational Medicine (EASOM) Statement on Work-Related Cancer

**DOI:** 10.3390/ijerph17072279

**Published:** 2020-03-28

**Authors:** Begoña Martínez-Jarreta, Nicole Majery, Petar Bulat, Soile Jungewelter, Elena-Ana Păuncu, Dieter Weigel, Marija Bubas, Alenka Škerjanc, Ivars Vanadzins, Maija Eglite, Marcos Larrosa, Susan Jill Stocks, Lode Godderis

**Affiliations:** 1 Occupational Medicine, Faculty of Medicine, University of Zaragoza and Scientific Research Group GIIS-063 (IIS-Aragón), 50009 Zaragoza, Spain; 2 Service du Santé au Travail, Multisectoriel (STM), 1630 Luxembourg, Luxembourg; nicole.majery@stm.lu; 3 Department of Occupational Health, Faculty of Medicine, University of Belgrade, 11000 Belgrade, Serbia; petar.bulat@med.bg.ac.rs; 4 Finnish Institute of Occupational Health (FIOH), FI-00250 Helsinki, Finland; soile.jungewelter@ttl.fi; 5 Discipline of Occupational Health, University of Medicine and Pharmacy “Victor Babes”, 300041 Timişoara, Romania; eapauncu@gmail.com; 6 Academy of Occupational Medicine and Public Health, 12159 Berlin, Germany; weigel-arbeitsmedizin@mailbox.org; 7 Croatian Institute of Public Health, 10000 Zagreb, Croatia; marija.bubas@gmail.com; 8 Clinical Institute of Occupational Medicine, University of Ljubljana, 1000 Ljubljana, Slovenia; alenka.skerjanc@gmail.com; 9 Institute of Occupational Safety and Environmental Health, Riga Stradins University, LV1007 Riga, Latvia; Ivars.vanadzins@rsu.lv; 10 Department of Occupational and Environmental Medicine, Riga Stradins University, LV1007 Riga, Latvia; maija.eglite@rsu.lv; 11 Internal Medicine Department, University Hospital Lozano Blesa, 50009 Zaragoza, Spain; mlarrosa@salud.aragon.es; 12 Health Services Research & Primary Care, Division of Population Health, University of Manchester, Manchester M13 9PL, UK; Jill.Stocks@manchester.ac.uk; 13 Centre for Environment and Health, KU Leuven, 3000 Leuven, Belgium; lode.godderis@kuleuven.be

**Keywords:** work related cancer, occupational cancer, prevention, education and training, EASOM

## Abstract

Reducing the burden of occupational cancers (OCs) is currently one of the most challenging Occupational Health (OH) issues. The European Union (EU) has made efforts to improve the existing legal framework and developed specific legislation aimed at reducing the burden of OC. However, available data suggest that OC are underreported. In August 2019, the European Association of Schools of Occupational Medicine (EASOM) adopted a statement that highlighted the importance of improving the education and training of Medical Doctors (MDs) to facilitate improvements in recognizing and reporting OC. To achieve this, EASOM proposes to promote OH education and training of MDs at undergraduate and postgraduate levels, foster harmonization of OH education and teaching standards and programs across EU countries, and enhance cooperation between universities and international scientific associations. Finally, we suggest that occupational data should be recorded in cancer and medical registers. By engaging MDs more fully in the debate about OCs, they will become more aware of the Occupational Physician’s role in reducing the burden of OCs and, furthermore, embed consideration of occupation as a potential cause of cancer into their own practice. These interventions will help promote the implementation of policies and interventions aimed to reduce OC in the workplace.

## 1. Introduction

Cancer is the leading cause of work-related deaths, and the International Labour Organization (estimated that over 100,000 cancer deaths in Europe in 2011 could be attributed to occupational causes [1]. A number of studies have estimated the population attributable fraction (PAF; proportion of cancer that could be avoided in the absence of the risk factor) in different countries, resulting in a wide range of estimates of the PAF, ranging from 3% to 10%, according to countries, cancers, and exposures [2,3]. In the occupational context, the PAF is the number of cases in a population with the occupational risk factor, minus the number of cases in the population without the occupational risk factor, divided by the total number of cases in the population. For example, PAF equals (O − E)/O, where O refers to the observed number of cases and E is the expected number of cases without exposure. Hence, PAF is a fraction that describes what proportion of cancer cases are caused by the exposure or risk factor if the exposure or risk factor causes the entirety of excess cancers [4,5]. Consequently, reliable information regarding the link between occupational exposures and cancer is rare, since occupation is rather rarely recorded. Even though there is knowledge of workplace exposure to carcinogens, there is a lack of empirical evidence about how this plays out in the workforce [5]. Besides, there is a wide difference regarding occupational exposure frequency among countries. Despite these caveats, we do have some reliable estimates of incidence and prevalence at the regional and country level that indicate the importance of occupation in causation of cancers [6,7].

Occupational cancer (OC) is not just a tragedy for the individual; in 2015 the direct costs of OC were estimated to be $6–$10 million Euros in the European Union [8].

Fortunately, there is a broad agreement that OC is largely avoidable. In Great Britain, 86% of work-attributable cancers are associated with exposure to 14 frequently used agents and occupational circumstances. Taking the most stringent measures to reduce these exposures could reduce cancers attributed to occupation from the current 10,000 or so per year to around 2000 per year by 2060 (assuming that the putative exposure is indeed causal) [9].

Given the magnitude—combined with a lack of visibility and awareness—of OC, the European Union (EU) and its member states have recently made additional efforts to improve the existing legal framework and develop specific legislation in an attempt to tackle OC. Other measures and initiatives have also been taken in different areas, such as the European campaign on dangerous substances by Occupational Safety and Health Administration (OSHA) from 2018 to 2019 and the Roadmap of Carcinogens initiated under the Dutch Presidency in 2016 [10]. EU Directive 2004/37/EC constitutes a cornerstone in protecting workers from exposure to carcinogens or mutagens at work, and has undergone significant updates, including Directive 2017/2398 of the EU Parliament, published on 12 December, 2017 and Directive 2019/983, published on 5 June, 2019 [11]. All of these legislative developments are an important step forward in promoting the protection of workers from risks, which arise or may arise from exposure to carcinogens and mutagens at work; although, “not all carcinogens or mutagens are covered” by these provisions [11,12].

In the field of occupational medicine, attention to OC is growing. For example, occupational cancer (OC) prevention was a key theme of the 32nd International Conference on Occupational Health (OH) held in Dublin in 2018, resulting in the adoption of a statement entitled “New Avenues for Prevention of Occupational Cancer and Other Severe Occupational Health Hazards” [13]. Further publications on this topic [14,15,16] have summarised the needs, and highlighted the crucial elements, to fight OC in the coming years.

It is indisputable that reducing OC depends on the success of primary prevention. This is made more challenging by the lag (often years) between exposure and cancer, but primary prevention should be seen as in investment for the future. Reductions in cancer incidence and mortality are highly cost effective, however the full benefit will only be realised decades after the introduction of primary prevention [16].

The case of asbestos is a good example. In the United Kingdom, preventative measures, such as the ban on import and use of asbestos were implemented in 1999, but a reduction in the incidence of mesothelioma was not expected before 2016 [17].

The primary prevention of OC requires multi-sectoral approaches and multiple partnerships. Collaboration is needed, both between and within health and non-health sectors (such as the environment, labour, housing, transport, industry, and trade), community organizations, private enterprises, health and workers’ compensation, insurance organizations, and other key actors at the national and international levels [16,18].

The role of the Occupational Health Physicians (OHP) in primary, secondary, and tertiary prevention includes reducing exposure to occupational carcinogens mainly through the activities listed below:

1. risk assessment, monitoring, and registration of worker’s exposure to carcinogens;
2. teaching and training workers in risk exposure and protective measures;
3. adequate interaction and communication with stakeholders, employers, hygienists, other occupational experts, trade unions, etc.;
4. attributability (diagnosis) of occupational cancers;
5. medical surveillance and follow-up of the exposed worker;
6. interaction, cooperation, and communication with other medical specialists involved in cancer treatment and rehabilitation.

Additionally OHPs are usually involved in deciding whether the cancer is occupational, rather than the primary diagnosis. In fact, most occupational diseases will present via a route other than occupation health, especially cancer, so OHPs need to work with other medical doctors (i.e., primary care doctors, pneumonologists, haematologists, dermatologists, etc.) to understand the complete picture of occupational health. This synergy between OHP and other medical doctors (MDs) would be strengthened by offering the opportunity for all physicians to gain skills and knowledge on occupational health and work related health problems, including OC. A broader awareness of a potential occupational aetiology, or the possibility of an occupational exposure in the origin of a disease, will emphasise the need for primary and secondary prevention (for those already exposed to occupational carcinogens and, therefore, at increased risk) more widely [13,15].

The European Association of Schools of Occupational Medicine (EASOM), established in 1993, promotes the highest standard of education and training in occupational medicine among health workers, especially MDs and OHP. EASOM dedicated its 19th Summer School in Riga (29–31 August 2019) to discuss strategies to address OC through education, awareness, and multi-sectoral collaboration. On 31 August, EASOM adopted the statement below.

EASOM statement on Occupational Cancer, Riga, 31 August 2019.

### 1.1. Investment in Education and Training of Medical Doctors is Required to Ensure That

- exposures to occupational carcinogens are recognised and reduced;
- secondary prevention is in place for exposed workers;
- cases of OC are recorded and reported accurately (discussed further in recommendation 4 below).

#### 1.1.1. Awareness and Knowledge about Occupational Cancer Must Be Increased in Order to

- foster a better understanding of the health risks caused by exposure to occupational carcinogens;
- improve compliance with preventive and protective measures in cooperation with other OH Professionals (hygienists, etc.) as well as ethical work practices [15].

#### 1.1.2. Training of Medical Students and Physicians in Occupational Medicine Should Be Provided throughout Their Medical Career

Currently, many medical students may graduate without having received specific training in Occupational Medicine (OM) compounded by little attention to occupational aetiology in the health problems that are studied in other subjects (e.g., dermatology, haematology, oncology, etc.). Many European medical schools do not include Occupational Medicine/Occupational Health at all, or if they do, only a few hours are devoted to this subject [19,20]. A general negative attitude toward OH accompanied by little knowledge of OH, and lack of awareness on occupational risks, has been described in medical students, which seems to be linked to their poor training [21,22]. However, training undergraduate medical students in OM has shown to raise awareness and bring about a positive and significant change of attitude towards occupational aetiology and work-related diseases [22]. 

We recommend that all physicians receive training in the basic knowledge and skills of OM, as well as integrated in to particularly relevant specialties, such as pneumonology, haematology, dermatology, etc., in medical school and throughout their medical careers, as part of continuing medical education accreditation programs [23]. Many exposures that may cause cancer in the long term are already relevant to specialists, such as pneumonologists or dermatologists, due to the short- term consequences of the exposure, e.g. chromate VI [24].

Both policy makers and those in medical education should take action to address these basic needs to engage all relevant professionals in reducing the burden of OCs.

#### 1.1.3. The Training of Specialists in OM Should Continue to Strive to Improve Standards and Ensure that OC Receives High Priority.

Considerable effort has been made to have a basic common curriculum to establish training standards and promote standardisation in OM medical specialist training within the European Union [25]. Recently, a European exam has been launched by UEMS-OM (Occupational Medicine Section of the European Union of Medical Specialists) and supported by EASOM to facilitate the necessary standardisation for the free movement of medical specialists within the EU. 

Competences on risk assessment, monitoring, and registration of worker’s exposure to carcinogens; teaching and training workers in risk exposure and protective measures; attributability (diagnosis) of OCs; medical surveillance and follow-up of the worker exposed to carcinogens, etc., are currently tested in OM specialist training exams.

#### 1.1.4. The Training of Primary Care Doctors and Other Medical Specialists Cannot Be Neglected

Primary care doctors are key professionals in detecting, diagnosing, and treating occupational diseases (including cancer), obliging them to not only provide appropriate care but to provide secondary prevention (extra screening), plus recognise and report occupational diseases (ODs). Adequate training for these tasks is essential [25,26]. We recognise that primary care doctors are constantly overburdened but, given the huge costs of OC, any investment would soon be repaid. Something as simple as including a section in clinical records concerning occupations and possible exposures to carcinogens would be a big improvement on the existing knowledge, allowing better monitoring for regulatory action and designing interventions [26,27,28].

The above should also apply in tertiary care to oncologists and other specialists (e.g., pneumonologists, haematologists) dealing with patients who have cancer. All medical specialists involved in the diagnosis, treatment, and rehabilitation of cancer patients should be aware of the importance of occupational aetiology, firstly to ensure that patients receive the compensation to which they are entitled (requiring a legally-defined level of evidence for causation) and, secondly, to make improvements in prevention strategies through the knowledge gained by surveillance and epidemiological study [29].

## 2. Adequate Education Opportunities and Training Are Crucial in Promoting Multi-Sectoral Collaboration regarding Occupational Diseases and Occupational Cancer

They contribute to:

### 2.1. Mutual Understanding and Collaboration among OH Professionals

The cooperation of different professionals, such as hygienists, engineers, psychologists, etc., working in government agencies, such as health, work, social security, environment, or industries such as agriculture or education, are essential to deliver high quality OH. A shared understanding and respect for each professional’s role and responsibilities is required for success. Furthermore, teamwork and engagement of all professionals in the process will foster job satisfaction and better outcomes.

### 2.2. Mutual Understanding and Collaboration among Occupational Physicians and Other Medical Specialists

An interdisciplinary approach rather than multidisciplinary is required. Cooperation among all medical specialities is crucial to tackle OCs [30].

## 3. Medical Schools, Universities, and International Scientific Associations Have a Crucial Role Concerning Recognition and Adequate Training in OH

University centres are connected internationally, and can cooperate in achieving essential goals, such as developing a standard basic curriculum in EU countries or establishing minimum training requirements in OH for health professionals and others involved in OH.

EASOM and other scientific societies must cooperate in this field and ensure that governments and policymakers understand the key role of the universities/academia in overcoming the current challenges that OH faces.

## 4. All Professionals Working with Access to Cancer Registry Reporting Systems Should Be Encouraged to Contribute Information to Cancer Registers

Substantial differences can be identified among European countries regarding cancer registries, particularly concerning the inclusion of occupation data in all EU countries cancer registries [31]. We need information about the exposures associated with OC if we are to reduce said exposures. As a minimum, the main occupation and employing industry of the patient should be reported to the cancer registry. Without improvements in recording occupational data in cancer registries, we will remain unaware of the size of the burden of OC and of new and/or changing occupational exposures associated with cancer. Better knowledge will allow us to target occupations needing preventative interventions within a shorter time scale and instigate secondary prevention more quickly. In short, there would be all the usual benefits associated with surveillance of any heath problem.

Generally, the harmonisation of cancer records across all EU countries and the inclusion of occupation data has to be a priority to estimate the burden of OCs and reduce the relevant exposures, but this is a matter of policy rather than physician training. However, all doctors should know the importance of occupational exposure data and be able to identify OCs. Engagement of all MDs will bring about changes in policy and co-operation recording ODs more quickly. 

## 5. Interventions Are needed to Implement Policies in to the Work Place

Current EU legislation has been proactive in its aim to eliminate OCs and other occupational diseases. Nevertheless, EU legislation does not guarantee the practical implementation of rules within the countries. Cultural and educational related factors can support and promote local opposition to the rules and obligations, thereby making them ineffective. Education and training initiatives should be part of any strategy to overcome local barriers to effective legislative implementation. They can contribute to optimised/adapted solutions to diversity between countries, such as the specifics of their labour market and social security system, etc. These education and training initiatives are urgently needed to reduce the gap between policies, legislative measures, and implementation in to the workplace for all ODs, including OCs.

### What EASOM Can Do

EASOM brings together academic centres, institutions, and schools of OM of 19 EU countries with substantial differences in their population structure, culture, wealth, and industrial development, health, and social security systems, and resources for Occupational Health.

One of EASOM’s primary objectives is to promote the highest standards in education and training in OM, thereby raising awareness about the importance of training in OCs, particularly aetiology, exposure, and prevention in OM, as well as in other specialities. To achieve this goal, EASOM will collaborate with other scientific associations to define the requirements in training of health professionals and MDs to promote better Occupational Health and to standardise high-quality training standards across all of these countries. Additionally, EASOM raises awareness about the key role of Occupational Physicians in reducing the burden of OCs and promotes the importance of this medical speciality.
